# Anti-Viral Activities of Umbilical Cord Mesenchymal Stem Cell-Derived Small Extracellular Vesicles Against Human Respiratory Viruses

**DOI:** 10.3389/fcimb.2022.850744

**Published:** 2022-04-21

**Authors:** Soo-Jin Oh, Eun-Na Lee, Joo-Hoo Park, Jae Kyung Lee, Geum Joon Cho, Il-Ho Park, Ok Sarah Shin

**Affiliations:** ^1^ BK21 Graduate program, Department of Biomedical Sciences, College of Medicine, Korea University Guro Hospital, Seoul, South Korea; ^2^ Department of Medicine, Korea University College of Medicine, Seoul, South Korea; ^3^ Department of Otorhinolaryngology-Head and Neck Surgery, Korea University College of Medicine, Seoul, South Korea; ^4^ Upper Airway Chronic Inflammatory Diseases Laboratory, Korea University, Seoul, South Korea; ^5^ Department of Obstetrics and Gynecology, College of Medicine, Korea University Guro Hospital, Seoul, South Korea

**Keywords:** small extracellular vesicles (exosomes), UCMSCs, respiratory viruses, anti-viral, ALI (Air-Liquid Interface)

## Abstract

The endemic and pandemic caused by respiratory virus infection are a major cause of mortality and morbidity globally. Thus, broadly effective antiviral drugs are needed to treat respiratory viral diseases. Small extracellular vesicles derived from human umbilical cord mesenchymal stem cells (U-exo) have recently gained attention as a cell-free therapeutic strategy due to their potential for safety and efficacy. Anti-viral activities of U-exo to countermeasure respiratory virus-associated diseases are currently unknown. Here, we tested the antiviral activities of U-exo following influenza A/B virus (IFV) and human seasonal coronavirus (HCoV) infections *in vitro*. Cells were subject to IFV or HCoV infection followed by U-exo treatment. U-exo treatment significantly reduced IFV or HCoV replication and combined treatment with recombinant human interferon-alpha protein (IFN-α) exerted synergistically enhanced antiviral effects against IFV or HCoV. Interestingly, microRNA (miR)-125b, which is one of the most abundantly expressed small RNAs in U-exo, was found to suppress IFV replication possibly *via* the induction of IFN-stimulated genes (ISGs). Furthermore, U-exo markedly enhanced RNA virus-triggered IFN signaling and ISGs production. Similarly, human nasal epithelial cells cultured at the air-liquid interface (ALI) studies broadly effective anti-viral and anti-inflammatory activities of U-exo against IFV and HCoV, suggesting the potential role of U-exo as a promising intervention for respiratory virus-associated diseases.

## Introduction

Respiratory viruses remain a significant global health risk due to the potential emergence of novel, highly virulent strains that can result in a pandemic. From the 1918 H1N1 “Spanish flu”, 1968 H3N2 “Hong Kong flu” and 2009 H1N1 “swine flu”, to the most recent severe acute respiratory syndrome coronavirus 2 (SARS-CoV-2), high transmissibility of respiratory viruses and rapid emergence of numerous variants have hindered our attempts to control the pandemic. Common respiratory viruses causing human infections include human seasonal coronavirus (HCoV) and influenza A/B virus (IFV). So far, four species of HCoV have been identified, including 2 alpha-coronaviruses (HCoV-NL63 and HCoV-229E) and 2 beta-coronaviruses (HCoV-OC43 and HCoV-HKU1). Of those, HCoV-OC43 are considered as an alternative source that provides insights into highly pathogenic coronaviruses such as Middle East respiratory syndrome coronavirus (MERS-CoV), severe acute respiratory syndrome coronavirus 1 (SARS-CoV-1), and SARS-CoV-2, as these viruses all belongs to the same beta-coronavirus genus (Li et al., 2020). HCoV causes acute respiratory infection with a variety of symptoms, although infections are usually followed by mild upper respiratory tract diseases, and no specific therapy is currently available to treat HCoV infections (Park et al., 2020). Meanwhile, IFV has been intensively investigated for many years. Despite extensive surveillance, mismatches of the annual influenza vaccines occur occasionally as a result of continuous genetic mutation (antigenic drift) and genetic reassortment (antigenic shift). Antiviral therapeutics have been developed for IFV, but the emergence of drug-resistant virus types limits their usage (Krammer et al., 2018; Lampejo, 2020). Therefore, broadly effective antiviral drugs with refined antiviral mechanisms are necessary to prepare for the next pandemic.

Exosomes are small extracellular vesicles (EVs) that are naturally secreted by most cell types, are less than 150 nm in diameter, and enriched in tetraspanins such as CD9, CD63 and CD81 and endosomal markers including Alix and TSG101. Once considered as cellular waste, exosomes were shown to have a key role in normal physiological processes including intercellular communication, immune regulation, and development (Jeppesen et al., 2019). Exosomes also function as a carrier of signaling contents including proteins, lipids, and microRNAs (miRNAs) that can cross biological barriers (Valadi et al., 2007). In addition, exosomes derived from mesenchymal stem cells (MSCs) recapitulate most of the effects of intravenously injected MSCs and are now regarded as a safer alternative to cell transplantation, which is associated with the risk of tumorigenesis and immune incompatibility (Wei et al., 2020). Accumulating evidence suggest that MSC-driven exosomes may provide a diverse array of strategies to combat viruses, such as hepatitis C virus (HCV) and IFV-H5N1 (Qian et al., 2016; Loy et al., 2019)

Umbilical cord mesenchymal stem cells (UCMSCs) are subsets of multipotent stem cells and considered a potentially ideal cell type for regenerative therapeutics. UCMSCs can be easily obtained in a non-invasive manner, and quickly scaled up to clinical use. The inherent properties of immunomodulation and regeneration ability have emphasized UCMSCs as a promising source of emerging therapeutic strategies. In particular, UCMSCs are known for their immunosuppressive properties including low expression of major histocompatibility complex class I molecule and costimulatory factors such as CD80 and CD86 (Tipnis et al., 2010). Recent studies indicate that exosomes derived from UCMSCs (U-exo) have a significant role in immune modulation, tissue regeneration and antimicrobial defense by directly modulating immune response and enhancing the proliferative potential of tissues (Hollweck et al., 2012; Monguio-Tortajada et al., 2017; Lv et al., 2020; Liu et al., 2021; Palma et al., 2021). However, the role of U-exo on respiratory viral infection and pathogenesis has not been fully investigated.

Airborne transmission of respiratory viruses like IFV and HCoV underlines the importance of nasal airway epithelial cells localized in the respiratory tract in connecting the host immune response to the external environment, as these cells are the first to encounter airborne microorganisms. Culture of primary human nasal epithelial cells (HNECs) differentiated at the air-liquid interface (ALI) can recreate, *in vitro*, the polarized, pseudostratified respiratory epithelium populated by cells including ciliated cells and goblet cells (Lee et al., 2005). HNEC culture at the ALI involves exposing the apical side to air while the basal surface of the cells is in contact with the culture medium. Although recent studies have introduced ALI culture system as a suitable model to examine viral infection and pathogenesis (Loo et al., 2020), the application of ALI culture model to evaluate the antiviral efficacy of candidate drugs or therapeutics has been limited.

In this study, we utilized well-differentiated cultures of HNECs at the ALI to evaluate the antiviral effects of U-exo against IFV and HCoV, in addition to demonstrating the promising potential of ALI culture models as a suitable platform to investigate novel therapeutics against emerging respiratory viruses.

## Materials and Methods

### Cells and Viruses

Umbilical cords were collected from healthy donors after obtaining necessary informed consent with relevant Institutional Review Board (IRB) guidelines and regulations, and all experimental protocols were approved by IRB from Korea University Guro Hospital (Seoul, Korea). Mesenchymal stem cells were isolated from the virus-free umbilical cords using an in-house established protocol (Seong et al., 2020). In brief, the cord was cleaned with phosphate-buffered saline (PBS) (Invitrogen), blood clots were removed, and the cord was dissected into smaller explants and placed on tissue culture dishes in Dulbecco’s modified Eagle’s medium (DMEM) (Corning Mediatech) supplemented with 10% fetal bovine serum (FBS) (Corning Mediatech) and 1% non-essential amino acids (Sigma). The cells were maintained in a humidified atmosphere with 5% CO_2_ at 37°C and fresh media were supplemented every other day.

Human lung adenocarcinoma cells (A549), human colorectal adenocarcinoma cells (HCT8) and human lung embryonic fibroblast cells (MRC-5) were obtained from the American Type Culture Collection. A549 and HCT8 cells were cultured in RPMI 1640 medium (Corning Mediatech) and MRC-5 cells were cultured in DMEM supplemented with 10% FBS, and 1% penicillin/streptomycin.

Human IFV A/H1N1, A/H3N2, and B/Yamagata strains were obtained from Korea Bank for Pathogenic Viruses (KBPV). Human influenza virus A/Puerto-Rico/8/34 (H1N1) PR8 lacking non-structural protein 1 (NS1) (IFV-PR8delNS1) was previously reported (Garcia-Sastre et al., 1998). For viral infection, A549 cells were washed with PBS and incubated for 1 h at 37°C with the virus diluted in infection media (RPMI 1640 medium containing 7.5% bovine albumin fraction V, 1mM HEPES, 2 μg/mL TPCK-trypsin and antibiotics) at the indicated multiplicity of infection (MOI). The inoculum was aspirated, and cells were incubated with serum free RPMI. The supernatants were collected to determine 50% tissue culture infectious dose (TCID_50_) to calculate viral titer. Briefly, MDCK cells seeded in 96-well plate were inoculated with diluted viral supernatants and incubated at 37°C for 1 h. Serum-free media was then added to cells, which were incubated until the cytopathic effect was visible. At 72 h post infection (hpi), cells were collected and stained with trypan-blue solution (Invitrogen). Viral titer was determined using the Spearman-Karber method and expressed as TCID_50_ units/mL.

Human seasonal beta-coronavirus (HCoV-OC43) and alpha-coronavirus (HCoV-229E) were obtained from KBPV and propagated in HCT8 and MRC-5 cells, respectively, in RPMI medium and DMEM supplemented with 10% FBS, and 1% penicillin/streptomycin.

### Exosome Purification and Characterization

#### Purification

UCMSCs were seeded in 3 T75 flasks with a medium containing exosome-depleted FBS (Thermo Fisher Scientific). After 48 h, 40 mL of culture supernatant was collected and centrifuged at 300 × g for 10 min to remove cellular debris and 0.22 μm filtration using syringe was used to discard small particles including apoptotic bodies. Subsequently, conditioned medium was collected and exosome was isolated using classical ultracentrifugation method (Patel et al., 2019) or Exo-spin precipitation and size exclusion chromatography purification method according to manufacturer’s instruction (Cell Guidance Systems). EVs released from UCMSCs were characterized according to the Minimal Information for Studies of Extracellular Vesicles guidelines developed by the International Society for Extracellular Vesicles in 2018 (Thery et al., 2018).

#### Characterization

The size distribution and concentration of EVs were determined by Nanoparticle Tracking Analysis (NTA) using NanoSight 300 (Malvern Panalytical). EVs were loaded into the sample chamber of an LM10 unit and video of each sample was recorded for 60 s three times, and analyzed with the NanoSight NTA 3.1 software. Data were expressed as mean ± SD of the three recordings.

The morphology of the EVs were assessed by using transmission electron microscopy (TEM). Briefly, 50 μl of the exosome suspension was diluted in 100 μl DPBS and mixed with 150 μl of 4% paraformaldehyde for fixation. 10 μl of this mixture was transferred to formvar/carbon-coated electron microscopy grids and incubated for 20 min under a heat lamp for dehydration and adhesion. The preparations were examined by TEM (HT7700, Hitachi Ltd) at an accelerating voltage of 200 kV.

The surface markers of the isolated EVs were detected by immunoblot analysis. The protein concentration of exosome was determined using a BCA Protein Assay Kit (Pierce). U-exo were prepared in exosome resuspension buffer (Thermo Fisher Scientific), subjected to SDS-PAGE and transferred onto polyvinylidene difluoride membranes. Membranes were then incubated with the primary antibodies overnight at 4°C. The following primary antibodies were used: Anti-Calnexin (CST2679, Cell Signaling Technology; 1:1000 dilution), Anti-Alix (ab117600, Abcam; 1:1000 dilution), Anti-TSG101 (28283-1-AP, Proteintech; 1:1000 dilution), Anti-CD9 (CST13174, Cell Signaling; 1:1000 dilution) and Anti-CD63 (ab59479, Abcam; 1:1000 dilution). After incubation with the HRP-conjugated anti-rabbit or anti-mouse IgG secondary antibodies (Cell Signaling Technology), the chemiluminescent signal was detected using Fusion Solo X (Vilber).

### Cell Viability Assay

A549 or HCT8 cells were seeded in 96-well plates. After 24 h, the medium was changed, U-exo (50 particles/cell) was added, and the incubation was continued for 8, 24, and 48 h at 37°C. After the incubation, 3-(4, 5-dimethylthiazolyl-2)-2,5-diphenyltetrazolium bromide (MTT) (Sigma-Aldrich) solution was added for 4 h followed by dimethyl sulfoxide treatment for 30 min. The optical density was then measured at 570 nm using a spectrophotometer. Cell survival was also monitored by CCK8 assay (Dojindo) as per manufacturer’s instructions. Control samples were set to 100% survival and all other samples were expressed relative to the control.

### Luciferase Reporter Assay

A549 cells were plated in 96-well plates and transiently transfected with a mixture of plasmids using Lipofectamine 2000 transfection reagent (Invitrogen) next day. Empty control plasmid was used to ensure that each transfection well contains the same amount of total DNA. The following luciferase reporter plasmids encoding *IFN-β, IFN-λ1, IFN-λ2* and *NF-κB* promoters were described in our previous study (Oh and Shin, 2021). At 24 h post-transfection, the luciferase activity was detected with Dual-glo luciferase reporter assay system (Promega) using Varioskan™ LUX multimode microplate reader (Thermo Scientific).

### miRNA Mimic Transfection

A549 cells were seeded in 12-well plates with 2% RPMI and transiently transfected with miRNA mimic control or miR-125b-5p specific mimic (200 pmol) using Lipofectamine RNAiMAX transfection reagent (Invitrogen). After 24 h, A549 cells were infected with IFV A/H1N1 at indicated MOI. At 48 hpi, RNA was extracted for cDNA synthesis and RT-qPCR. miR-125b-5p mimic sequence are as follows: UGCCUGAGACCCUAACUUGUG.

### Reverse Transcription-Quantitative PCR

Total RNA was extracted using Direct-zol RNA mini Prep Kit (Zymo Research) and was reverse transcribed to generate cDNA using the Reverse Transcription system (Promega) for 1 h at 42°C. RT‐qPCR assays are performed using two-step approaches, cDNA synthesis followed by qPCR. Using a Power SYBR Green PCR Master Mix (Thermo Fisher Scientific), viral and host gene expression levels were measured. Primer sequences for host genes were previously reported (Oh et al., 2021) and viral genes are as follows. *IFV-A NP F: CTCGTCGCTTATGACAAAGAAG, IFV-A NP R: AGATCATCATGTGAGTCAGAC, IFV-A M1 F: AAGACCAATCCTGTCACCTCT GA, IFV-A M1 R: CAAAGCGTCTACGCTGCAGTCC, IFV-A M2 F: CCGAGGTCGAAACGCCTATC, IFV-A M2 R: CTTTGGCACTCCTTCCGTAG IFV-B M1 F: GAGACACAATTGCCTAC-CTGCTT, IFV-B M1 R: TTCTTTCCCACCGAACCAAC, HCoV NP F: CCTTCCTGAGCCTTCAA-TATAGTAACC, HCoV NP R: ACGTACTTCTATTATGAAGCATGA-TATTAA.* The cycling parameters were as follows: 95°C for 15 min, followed by 40 cycles of 30 s at 95°C and 1 min at 60°C. *Glyceraldehyde 3-phosphate dehydrogenase* (*GAPDH*) or *18s rRNA* was used as a reference gene.

### Immunoblot Analysis

Immunoblot analysis were performed as described previously (Lee et al., 2021). Cells were harvested and lysed with RIPA buffer (Sigma) containing protease and phosphatase inhibitors (Roche). Proteins were separated by SDS-PAGE, transferred onto PVDF membranes, and blocked with 5% skim milk in TBS supplemented with 0.1% Tween-20 (TBS-Tw) for 1 h at room temperature. The membranes were then incubated with primary antibodies (Cell Signaling Technology, 1:1000 dilution) at 4°C overnight, followed by HRP-conjugated anti-rabbit or anti-mouse IgG secondary antibodies (Cell Signaling Technology) for 1 h at room temperature. Anti-β-actin (Abgent) antibody was used as a loading control.

### Confocal Microscopy

HCT8 cells were seeded onto coverslips in 24-well plates. The next day, cells were inoculated with HCoV-OC43 and then treated with U-exo (50 particles/cell) for 5 days. After washing with PBS, the cells were fixed with 4% paraformaldehyde and permeabilized with 0.1% Triton X-100. Cells were blocked using 2.5% BSA in PBS solution and immunostained with 0.5% BSA in PBS containing anti-OC43 primary antibody (MAB9012, Merck, 1:1000 dilution), followed by an anti-mouse Alexa 594 antibody. Next, cells were mounted onto glass slides using mounting media containing 4,6-diamidino-2-phenylindole (DAPI) (Vector Laboratories), which were examined by confocal microscopy (LSM900; Carl Zeiss).

### Characterization of Human Nasal Epithelial Cell Differentiation at Air-Liquid Interface (ALI)

Human nasal epithelial cells (HNECs) were collected from patients who underwent trans-sphenoidal pituitary tumor surgery. The study was approved by Korea University Medical Center Institutional Review Board (approval number: 2020GR0308) and written informed consent was obtained from all patients in accordance with the Declaration of Helsinki. Cells were collected by scraping the mid-inferior turbinate with a brush and cultured in PneumaCult™-Ex Medium (Stemcell). HNECs were amplified, washed with pre-warmed DPBS, and harvested using ACF Enzymatic Dissociation Solution (Stemcell). For the ALI culture system, the appropriate number of HNECs were seeded on apical chamber of 0.4 μm transwell membrane inserts (Corning Mediatech) with 0.5 mL PneumaCult™-Ex medium (Stemcell) and 1 ml PneumaCult™-Ex Medium was added to the basal chamber. The cells were incubated at 37°C and media in both the basal and apical chambers were changed every other day for 2-4 days. When the cells reached 100% confluence, the medium was removed from both basal and apical chambers. 1 mL PneumaCult™-ALI Maintenance Medium (Stemcell) was added only to the basal chamber and changed every 2 days.

After 3 weeks, the trans-epithelial electrical resistance (TEER) was measured to confirm the differentiation status of the ALI culture. To determine the TEER, cells cultured at the ALI were washed with pre-warmed DPBS to remove excessive mucus and measurements were carried out using a STX-2 chopstick electrode attached to an epithelial voltmeter (World Precision Instruments). ALI culture were fixed with 4% paraformaldehyde, and dehydrated in gradient alcohol series before they were embedded in paraffin. Sections of 4-μm thickness were employed for hematoxylin and eosin (H&E) staining. The morphology of cells was observed under Olympus BX51 light microscopy (Olympus). To confirm mucociliary clearance, Particulate matter (<10 μm, PM10) was added to surface of epithelial cells at upper chamber of ALI. Images of the cells were captured every 10 seconds using an Olympus BX71 microscope (Olympus). The ability of mucociliary clearance was confirmed by measuring the distance between the starting point of PM10 and the end point of PM10 after movement.

### Virus Infection of ALI Culture Cells

For apical infection, fully differentiated primary ALI culture was inoculated with IFV A/H1N1 and HCoV-OC43 in the presence or absence of U-exo or IFN-α. A mixture of virus and U-exo was added to the apical chamber and U-exo or IFN-α-containing media was supplemented in the basolateral side. Cells at ALI culture were incubated at 37°C and 5% CO_2_ for 90 min followed by removal of the virus from the apical chamber. The infected cells of the ALI culture model were subject to further analysis to determine the antiviral effects of U-exo treatment. To measure viral gene expression, total RNA from ALI culture were extracted using Direct-zol RNA mini Prep Kit (Zymo Research). To measure the effect of U-exo on cell death caused by viral infection, extracellular release of lactate dehydrogenase (LDH) was measured using a CytoTox96 non-radioactive cytotoxicity assay kit (Promega) according to manufacturer’s instructions. LDH release was calculated as [extracellular LDH/(intracellular LDH + extracellular LDH) × 100].

### Immunofluorescence Staining of ALI Culture Cells

Cells cultured at the ALI were fixed with 4% paraformaldehyde and permeabilized using 0.1% Triton X-100 in PBS. The cells were blocked with 2.5% BSA in PBS for 20 min and incubated with the indicated primary antibodies (E-Cadherin, Santa Cruz, 1:200; MUC5AC, Santa Cruz, 1:200, ZO-1, Invitrogen, 1:200; α-tubulin, Santa Cruz, 1:200) at 4°C overnight followed by incubation with a fluorescent secondary antibody (anti-Rabbit Alexa 594 conjugated antibody, Invitrogen, 1:200; anti-mouse FITC conjugated antibody, Invitrogen). The cells were stained with DAPI and the images were visualized using confocal microscopy (LSM900; Carl Zeiss).

### Enzyme-Linked Immunosorbent Assay

Supernatants from the basolateral side of ALI culture were collected to measure the secretion level of cytokines. IL-6, IL-8, MCP-1, and IFN-β ELISA kits were purchased from R&D Systems. Assays were performed according to manufacturer’s instructions. Absorbance at 450 nm was measured using a microplate spectrophotometer.

### Statistical Analysis

Statistical comparisons between the different treatments were performed using an unpaired two-tailed student’s t-test or Mann Whitney test (Graphpad Prism) and p < 0.05 was considered statistically significant.

## Results

### Characterization of UCMSC-Derived Small Extracellular Vesicles (U-exo)

**Figure 1:**
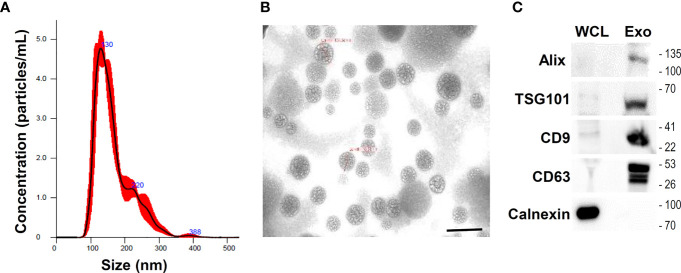
Isolation and characterization of umbilical cord mesenchymal stem cells (UCMSCs)-derived exosomes (U-exo). UCMSCs were cultured in medium containing exosome-depleted FBS for 48 h and culture supernatant was collected and centrifuged at 300 × g for 10 min to remove cellular debris and 0.22 μm filtration using syringe was used to discard small particles including apoptotic bodies. Subsequently, conditioned medium (CM) was collected and exosome was isolated using Exo-spin precipitation and size exclusion chromatography purification method. **(A)** Nanoparticle tracking analysis of exosomes isolated and purified from UCMSCs. The mean diameter of the isolated exosomes was measured to be under 150 nm, indicative of the majority of extracellular vesicles (EVs) being exosomes. **(B)** Transmission electron microscopy of U-exo at 70,000x magnification. Scale bar =200 nm **(C)** Immunoblot analysis of U-exo markers (Alix, TSG101, CD9, and CD63) is shown. Calnexin (Endoplasmic reticulum marker) was used as a negative control. WCL: whole cell lysates; Exo: U-exo.

Conditioned medium (CM) from UCMSCs was collected for the isolation and purification of exosomes. Particles in the CM were enriched by using ultrafiltration with a cut-off of 10 kDa. U-exo was further identified by size distribution, concentration, and morphology. NTA of the small extracellular vesicles isolated from UCMSCs indicated that 4.24 x 10^10^ particles/mL were isolated with a mean particle size of 137.5 nm (**Figure 1A**). The morphology and size of the U-exo were also visualized using TEM (**Figure 1B**). Furthermore, U-exo were shown to express Alix, TSG101, CD9, and CD63, all of which are common EV markers that are either involved in EV biogenesis or expressed on the surface of EVs (**Figure 1C**). Calnexin, an endoplasmic reticulum marker used as a negative marker to confirm successful exosome isolation and purification, was only detected in the whole cell lysates, suggesting that the exosomes were not contaminated with cellular debris. We also prepared U-exo using classical ultracentrifugation method and the result was comparable in recovering exosomes ranging in size 50 to 150 nm. (**Supplementary Figures 1A, B**). These data show that U-exo was successfully isolated for use in subsequent experiments.

### Antiviral Activities of U-exo Are Broadly Effective Against Respiratory Viruses *In Vitro*

Cell cytotoxicity of U-exo on A549 cells and HCT8 cells was evaluated before studying the antiviral effects on IFV and HCoV. A549 cells and HCT8 cells were exposed to U-exo for 8, 24, and 48 h, respectively. Both CCK8 and MTT assays demonstrated that treatment with U-exo did not cause cytotoxicity in A549 and HCT8 cells (**Supplementary Figure 2**). For subsequent experiments, we used 50 nanoparticles per one cell.

**Figure 2:**
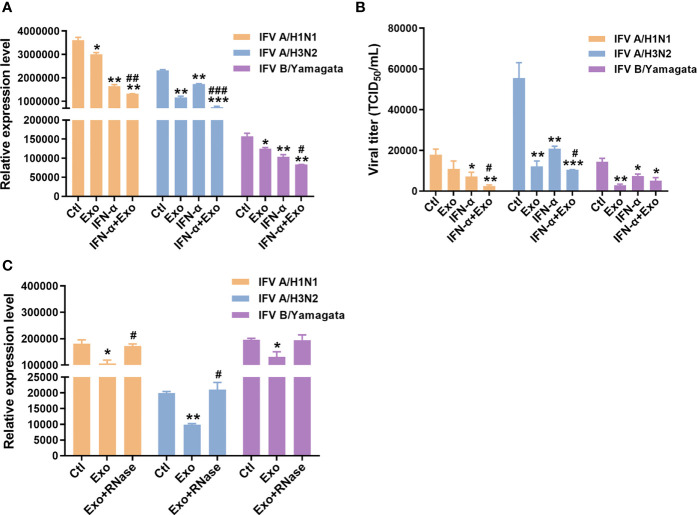
U-exo treatment reduces IFV replication and shows a synergistic antiviral effect with IFN-α on IFV infection. **(A)** A549 cells were infected with IFV A/H1N1, IFV A/H3N2 and IFV B/Yamagata at an MOI of 1. After viral attachment, cells were treated with control (Ctl), U-exo (Exo), IFN-α (10 ng/mL), or IFN-α combined with U-exo (IFN-α+Exo) for 48 h, after which viral gene expression levels were measured using RT-qPCR. Expression levels of the viral *nucleoprotein* (*NP*) for IFV A/H1N1 or H3N2 or *matrix protein 1* (*M1*) for IFV B/Yamagata were quantified and normalized against *GAPDH*. Data are shown as the mean ± standard deviation (SD) of the means from three independent experiments. **(B)** Viral titers were measured using the TCID_50_ assay and expressed as TCID_50_/mL (means ± SD; n = 3). Statistical analysis: *p < 0.05; **p < 0.01; ***p < 0.001, versus Ctl-treated cells. ^#^p < 0.05; ^##^p < 0.01; ^###^p < 0.001, versus IFN-α-treated cells. **(C)** A549 cells were infected with IFV A/H1N1, IFV A/H3N2 and IFV B/Yamagata at an MOI of 1. U-exo were exposed to 1 μg/mL RNase for 1 h and added to IFV-infected cells. Viral gene expression levels were measured (means ± SD; n = 3). Statistical analysis: *p < 0.05; **p < 0.01; versus Ctl-treated cells. ^#^p < 0.05, versus U-exo (Exo)-treated cells.

IFN-α is a universally expressed cytokine that induces an antiviral state of cells and is used as a first-line antiviral agent for viral infections including Hepatitis B virus (HBV) and HCV (Platanias, 2005). Therefore, we evaluated whether the combined treatment of U-exo and IFN-α has a synergistic inhibitory effect on viral infection. The antiviral effect of U-exo on IFV and HCoV was tested using A549 cells and HCT8 cells, respectively. A549 cells were infected at a MOI of 1, with IFV A/H1N1, IFV A/H3N2 and IFV B/Yamagata. After infection, cells were incubated with IFN-α in the presence or absence of U-exo. Effects on viral gene expression or viral titer were determined using RT-qPCR and TCID_50_ assay, respectively. Viral *nucleoprotein (NP), Matrix protein 1 (M1)* and *Matrix protein 2 (M2)* mRNA levels were significantly reduced in IFV A/H1N1 or IFV A/H3N2 or IFV B/Yamagata-infected cells, following U-exo treatment (**Figure 2A** and **Supplementary Figure 3A**). Viral titers were also significantly attenuated upon U-exo treatment, as determined by TCID_50_ assays (**Figure 2B**). As shown in **Figures 2A, B**, IFN-α exerted an inhibitory effect on all IFV strains, data which is consistent with previous findings (Wu and Metcalf, 2020). Furthermore, for each virus type, a significant reduction in viral gene expression levels and viral titer was observed when U-exo was combined with IFN-α, demonstrating the synergistic antiviral effect of U-exo and IFN-α. Of note, we also tested if small EVs (sEVs) isolated by the ultracentrifugation method would have a similar effect on viral load as sEVs purified using the kit-based method. Our data indicate that U-exo isolated by ultracentrifugation led to significant attenuation of viral gene expression in response to IFV A/H1N1 infection (**Supplementary Figure 1C**).

Exosomes have been reported to exert therapeutic effects mainly by delivering small RNAs to the target cells (Qian et al., 2016). To determine the potential antiviral role of small RNAs within the U-exo, we incubated U-exo with RNase to remove any RNA present and the mixture was added to cells. The RT-qPCR result showed that the U-exo was no longer able to inhibit IFV gene expression upon RNase treatment, suggesting the potential antiviral action of exosomal RNA content (**Figure 2C**).

**Figure 3:**
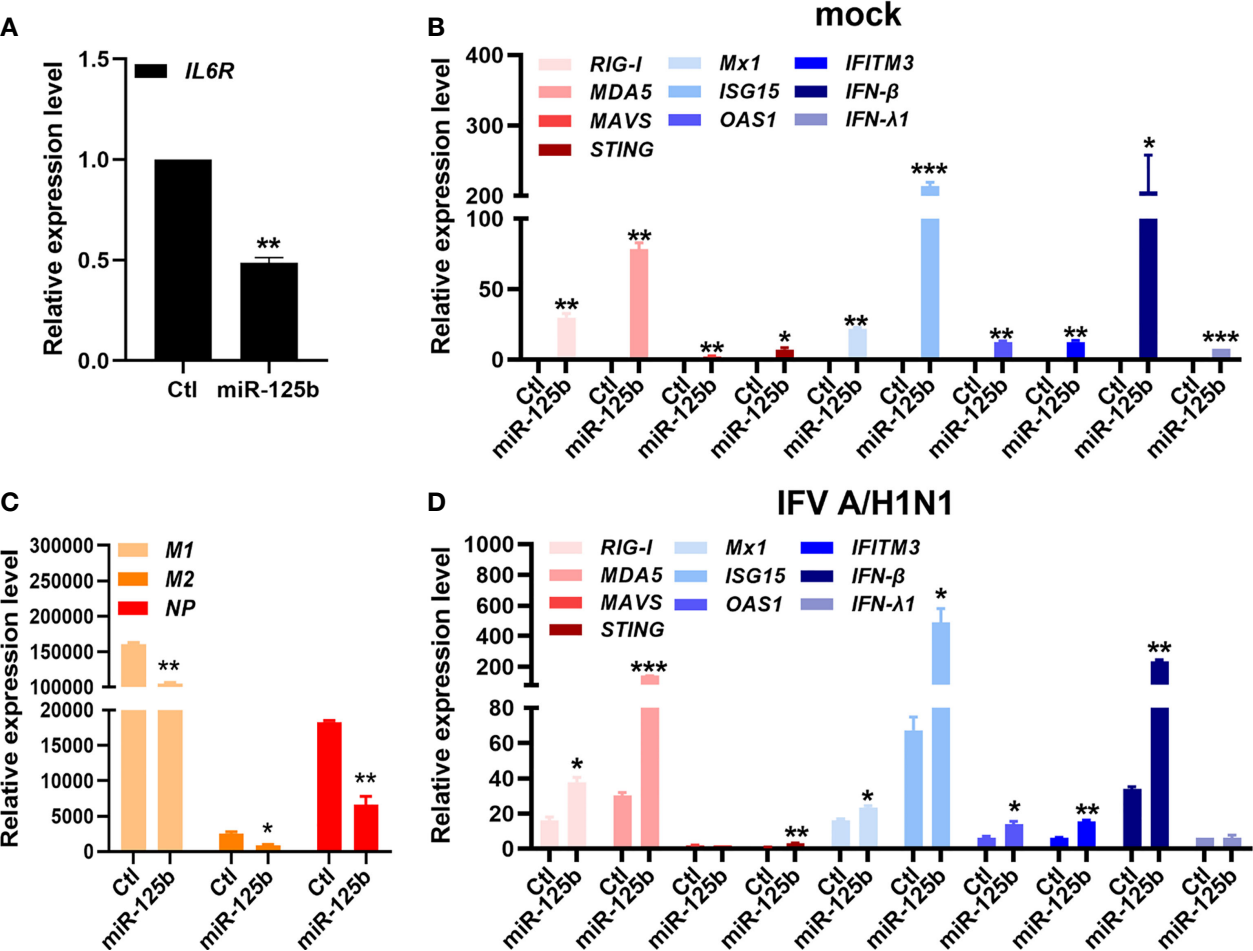
miR-125b-5p, one of the highly expressed microRNAs (miRNAs) in U-exo, is capable of antiviral action against IFV. A549 cells were transfected with control mimic (Ctl) or miR-125b-5p mimic for 24 hours. The next day, cells were infected with mock or IFV A/H1N1 at an MOI of 1 and RNA was collected 48 h later. **(A)** The effect of miR-125-5p mimic was verified by quantitative measurement of *IL-6R*, which is previously shown to be a target of miR-125-5p. **(B)** *Matrix protein 1* (*M1*), *matrix protein 2* (*M2*) and *nucleoprotein* (*NP*) expression levels were quantified and normalized against *GAPDH*. **(C, D)** Host gene expressions (*retinoic acid-inducible gene-I (RIG-I), melanoma differentiation associated protein 5 (MDA5), mitochondria antiviral signaling protein (MAVS), Stimulator Of Interferon Response (STING), MX Dynamin Like GTPase 1 (Mx1), Interferon stimulated gene 15 (ISG15), O2’-5’-Oligoadenylate Synthetase 1 (OAS1), Interferon Induced Transmembrane Protein 3 (IFITM3), interferon beta (IFN-β), and interferon lambda 1 (IFN-λ1)*), were also determined by RT-qPCR (means ± SD; n = 2). *p < 0.05, **p < 0.01, and ***p < 0.001, when compared to Ctl.

Considering that the presence of small RNAs in sEVs may affect antiviral activities, we further analyzed whether specific exosomal miRNAs are able to provide antiviral function against IFV. We previously reported differentially expressed exosomal small RNA profiles, which is deposited in Gene Expression Omnibus (GSE195634). According to this data, miR-125b-5p is one of the top differentially regulated miRNAs in U-exo. Therefore, we investigated whether miR-125b-5p would similarly exert an antiviral therapeutic effect. We transiently transfected A549 cells with control miRNA mimic or miR-125b-5p specific mimic. 24 h later, cells were infected with IFV and viral and host gene expression were determined following the infection. As shown by **Figure 3A**, we first confirmed that miR-125b-5p was able to effectively target *IL6R*, which has been previously shown by other groups (Yao et al., 2021). Furthermore, miR-125b-5p mimic treatment resulted in diminished expression of viral genes (*M1, M2, NP*), whereas it had an opposite effect on host genes, upregulating RIG-I-like receptor (RLR)-mediated antiviral gene expressions (**Figures 3B–D**). Thus, this data indicates the potential role of exosomal miRNAs, such as miR-125b-5p, acting as a key player of U-exo’s antiviral properties.

**Figure 4:**
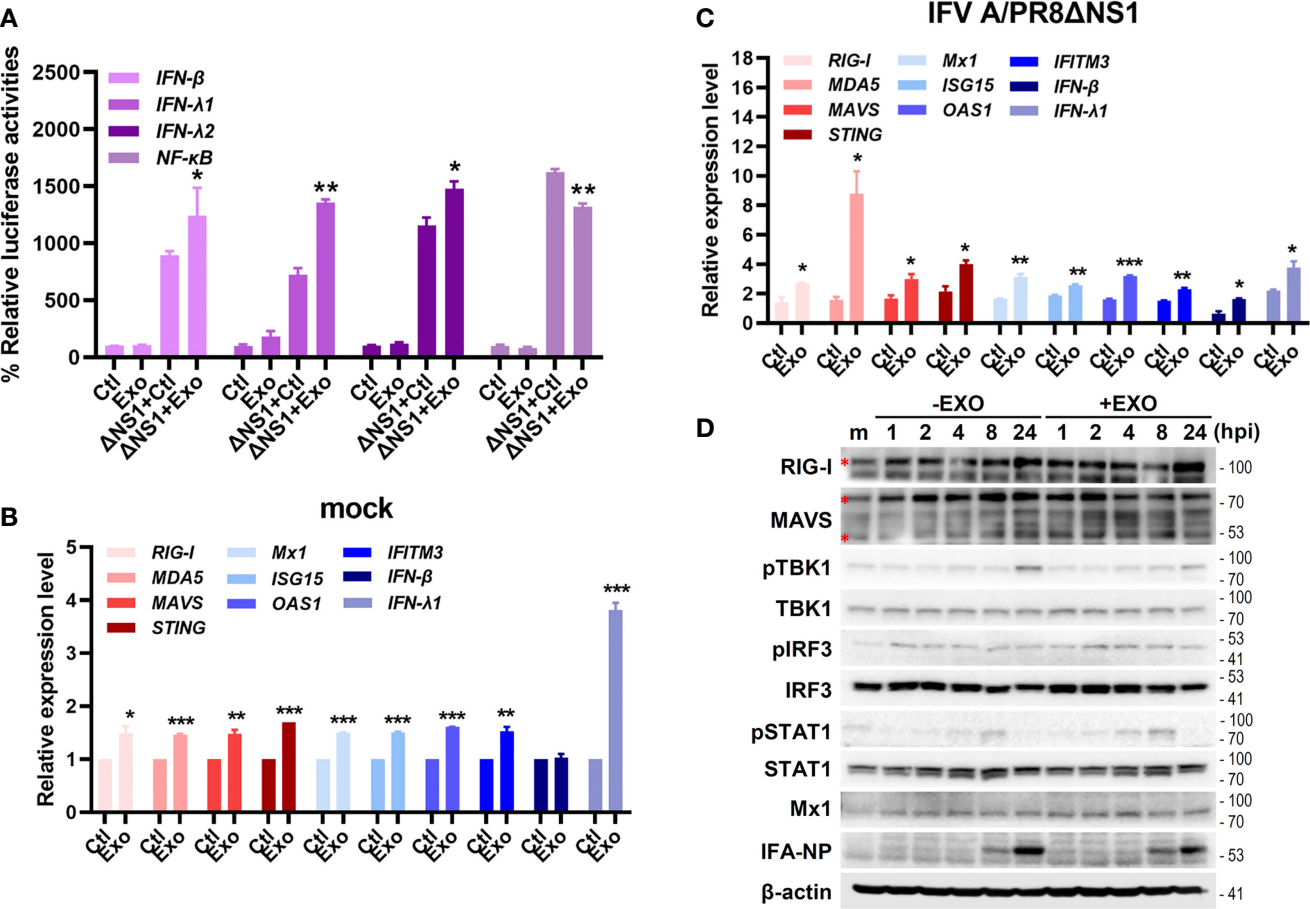
U-exo treatment enhances antiviral interferon (IFN)-mediated signaling and IFN-stimulated genes (ISGs) expression. **(A)** A549 cells were transfected with the indicated promoter (*IFN-β, IFN- λ1, IFN- λ2* and *NF-κB*)-driven firefly luciferase plasmids and a constitutively active Renilla luciferase construct (pRL-TK) for 24 h, followed by the infection with mock or IFV A/PR8delNS1 at an MOI of 1. Six hours later, cell lysates were harvested and subjected to luciferase assay. Values are expressed as mean of two independent experiments. **(B, C)** A549 cells were infected with mock **(B)** or IFV A/PR8delNS1 **(C)** at an MOI of 1. After viral attachment, cells were treated with control (Ctl), U-exo (Exo) for 4h, after which host gene expression levels were measured using RT-qPCR. Data are shown as the mean ± standard deviation (SD) of the means from two independent experiments. *p < 0.05; **p < 0.01; ***p < 0.001, vs. Ctl-treated cells. **(D)** The protein levels of RIG-I, MAVS, phospho-TBK/total TBK, phospho-IRF3/total IRF3, phospho-STAT1/total STAT1, Mx1 and IFV A NP were measured by immunoblot analysis.

To examine how U-exo exerts their antiviral therapeutic effect, we further investigated U-exo-mediated host cellular response to IFV infection. Of note, for subsequent experiments, IFV A/PR8delNS1 was used, considering that wildtype IFV is known to evade innate immunity *via* NS1 (Jia et al., 2010). A549 cells were transiently transfected with the following luciferase plasmids encoding promoters of *IFN-β, IFN-λ1, IFN-λ2* and *NF-κB* and 24 h later, cells were exposed to IFV A/PR8delNS1 (MOI 1). As shown in **Figure 4A**, U-exo treatment significantly augmented IFV A/PR8delNS1-induced induction of the *IFN-β, IFN-λ1, IFN-λ2* and *NF-κB* promoter activities mediated by RIG-I activation in A549 cells. To determine whether U-exo would affect the virus-triggered activation of IF Ns and interferon-stimulated genes (ISGs), A549 cells were infected with IFV A/PR8delNS1 and mRNA levels of antiviral immunity-related genes were measured by RT-qPCR. Expression of the following genes, *retinoic acid-inducible gene-I (RIG-I), melanoma differentiation associated protein 5 (MDA5), mitochondria antiviral signaling protein (MAVS), stimulator of interferon response c-GAMP interactor 1 (STING), Mx dynamin Like GTPase 1 (Mx1), interferon stimulated gene 15 (ISG15), O2’-5’-oligoadenylate synthetase 1 (OAS1), interferon induced transmembrane Protein 3 (IFITM3), interferon beta (IFN-β)*, and *interferon lambda 1 (IFN-λ1)*, were significantly elevated by U-exo treatment in both mock and IFV A/PR8delNS1-infected cells, compared with control-treated cells (**Figures 4B, C**). Lastly, we evaluated whether U-exo treatment increases ISGs production *via* upregulation of RLR signaling pathways. As expected, viral NP protein expression was greatly suppressed by U-exo treatment. On the other hand, U-exo treatment led to elevated protein levels of RLRs, such as RIG-I and MAVS, or downstream molecules of RLRs, such as phospho-TBK1, phospho-IRF3, and phospho-STAT1, in response to IFV infection (**Figure 4D**). Moreover, the induction of ISGs (Mx1) was also upregulated upon U-exo addition in A549 cells. Taken together, these findings indicate the possibility that U-exo-triggered IFN signaling and activation contribute to controlling viral replication.

**Figure 5:**
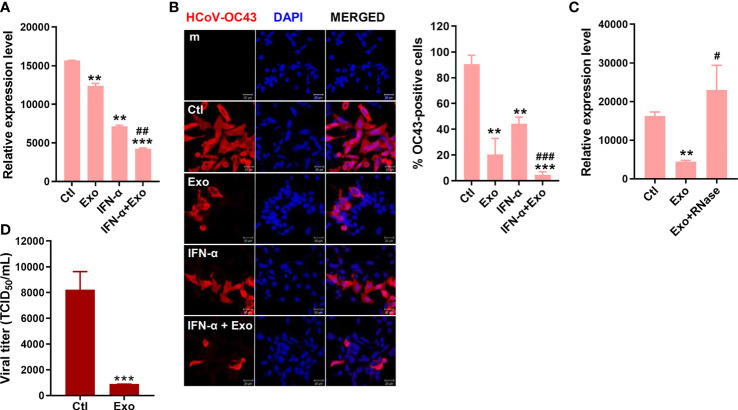
U-exo treatment reduces HCoV replication and shows a synergistic effect with IFN-a on HCoV infection. **(A)** HCT8 cells were infected with HCoV-OC43 at an MOI of 1. After viral attachment, cells were treated with control (Ctl), U-exo (Exo), IFN-α (10 ng/mL), or IFN-α combined with U-exo (IFN-α+Exo) for 5 days, after which viral gene expression levels were measured using RT-qPCR. Transcriptional expression of the viral *nucleoprotein* (*NP*) was quantified and the expression levels of viral genes were normalized to *GAPDH*. (mean ± SD; n = 3). **(B)** Immunofluorescent staining of HCoV-OC43 at 5 days post infection was visualized by confocal microscopy. Scale bar represents 20 μm. % OC43-stained cells were calculated and the graph shows an average of three independent experiments. Statistical analysis: **p < 0.01; ***p < 0.001, versus Ctl-treated cells. ^##^p < 0.01; ^###^p < 0.001, versus IFN-α-treated cells. **(C)** U-exo were exposed to 1 μg/mL RNase for 1 h and added to cells. Viral *NP* expression was measured (mean ± SD; n = 3). Statistical analysis: **p < 0.01; versus Ctl-treated cells. ^#^p < 0.05, versus U-exo (Exo)-treated cells. **(D)** MRC-5 cells were infected with alpha-coronavirus (HCoV-229E) (MOI 1) for 5 days. Supernatant was collected for TCID50 assay. Viral titers were measured using the TCID50 assay and expressed as TCID50/mL (means ± SD; n = 3). Statistical analysis: ***p < 0.001; compared with Ctl-treated cells.

The antiviral effect of U-exo on HCoV was also investigated. HCT8 cells were infected at a MOI of 1 with HCoV-OC43, washed, and incubated with U-exo. The expression levels of viral *NP* in OC43-infected cells were significantly reduced upon U-exo treatment at 5 dpi (**Figure 5A**). Immunofluorescence staining of OC43 virus confirmed that U-exo treatment suppressed the number of OC43-infected cells (**Figure 5B**). The synergistic effect of U-exo and IFN-α was shown by the significantly enhanced antiviral activities of IFN-α, determined by both RT-qPCR and confocal microscopy (**Figures 5A, B**). These findings were consistent with the results obtained using IFV. Furthermore, the antiviral effect of U-exo was diminished upon removal of RNA species by RNase treatment during HCoV-OC43 infection (**Figure 5C**). Also, we tested the effect of U-exo on alpha-coronavirus (HCoV-229E) infection. As shown by **Figure 5D**, U-exo treatment significantly attenuated HCoV-229E viral titer.

### U-exo Is Anti-Viral and Anti-Inflammatory During Respiratory Viral Infection in ALI Culture Model

**Figure 6:**
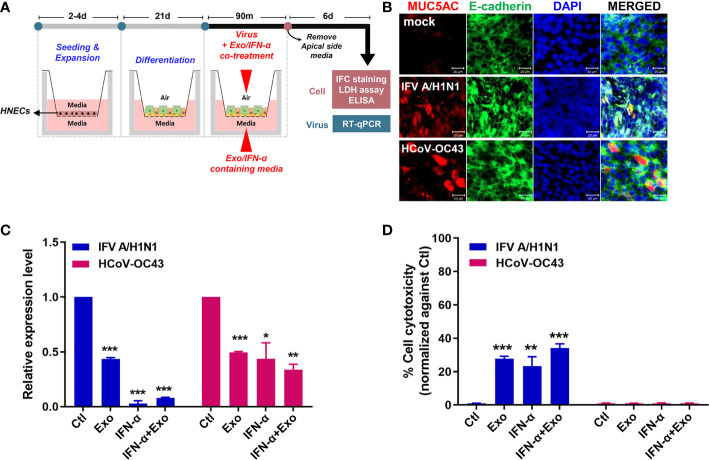
U-exo treatment in human nasal epithelial cells (HNECs) cultured at the air-liquid interface (ALI) exhibits an antiviral effect. **(A)** A schematic representing the experimental design. The differentiation status of the HNECs cultured at the ALI was confirmed prior to their infection with IFV A/H1N1, or HCoV-OC43. The infected cells of the ALI culture model were subject to further analysis to determine the antiviral effects of U-exo treatment. **(B)** HNECs cultured at the ALI were infected with IFV A/H1N1, or HCoV-OC43 viruses (MOI= 10). Structural integrity of the epithelium was evaluated by staining E-cadherin (green) and goblet cells were visualized by MUC5AC (red) staining. Scale bar = 20 μm. **(C)** Apical side was infected with IFV A/H1N1, or HCoV OC43 viruses (MOI= 10) at the same time as treatment with control (Ctl), U-exo (Exo), IFN-α (10 ng/mL), IFN-α in combination with U-exo (IFN-α+Exo). After 90 min incubation, media was removed from the apical side, and media containing U-exo and/or IFN-α was added to the basolateral side. Viral gene expression levels were determined after 6 days using RT-qPCR. *p < 0.05; **p < 0.01; ***p < 0.001, versus Ctl-treated cells. **(D)** The supernatants from the basolateral side were harvested to measure the extracellular release of lactate dehydrogenase (LDH) levels. Data are shown as the mean ± standard deviation (SD) of two independent experiments. **p < 0.01; ***p < 0.001, versus Ctl-treated cells.

To recapitulate the human airway epithelium, culture of primary HNECs were expanded, differentiated and cultured at ALI for 21 days to examine the antiviral and immunomodulatory effects of U-exo. The differentiation status of HNECs cultured at the ALI was confirmed by H&E staining (**Supplementary Figure 4A**). In particular, morphological analysis revealed the formation of an intact epithelial cell layer on the membrane as seen by H&E staining. Cilia formation and movement were confirmed and an immunofluorescence assay was used to determine the expression of ciliated cells (**Supplementary Figures 4B, C**). For viral infection, a mixture of virus and U-exo and/or IFN-α was added to the apical side and U-exo and/or IFN-α -containing media was supplemented to the basolateral side (**Figure 6A**).

First, we characterized the IFV A/H1N1-, or HCoV-OC43-infected HNECs at 6 dpi with immunofluorescence assay. The effect of the virus on the structural integrity of the barrier was determined by the distribution of E-cadherin, a commonly used marker for tight junctions. Moreover, since the mucosal barrier plays a critical role in the innate immune system of the lung, we analyzed the influence of infection on the major secreted mucin, MUC5AC. Regardless of virus types, virus infection resulted in disruption and discontinuation of E-cadherin, indicating a damage to the tight junction and structural integrity of the epithelium. Also, MUC5AC secretion was shown to increase upon virus infection (**Figure 6B**).

The antiviral effect of U-exo in ALI culture was investigated by measuring viral gene expression and the extent of cell cytotoxicity with RT-qPCR and LDH assay, respectively. HNECs cultured at the ALI were infected with MOI 10 of IFV A/H1N1, or HCoV-OC43, and were incubated with U-exo and/or IFN-α. **Figure 6C** demonstrates that U-exo treatment significantly reduced viral gene expression whereas IFN-α alone treatment completely blocked viral gene expression. Furthermore, IFV A/H1N1- infected HNECs that were treated with U-exo or IFN-α showed significantly lower levels of cell cytotoxicity. In contrast, no reduction in the level of cell death was observed following HCoV-OC43 infection, an expected finding as HCoV-OC43 rarely induces significant cytopathic effects (**Figure 6D**) (Dijkman et al., 2013).

**Figure 7:**
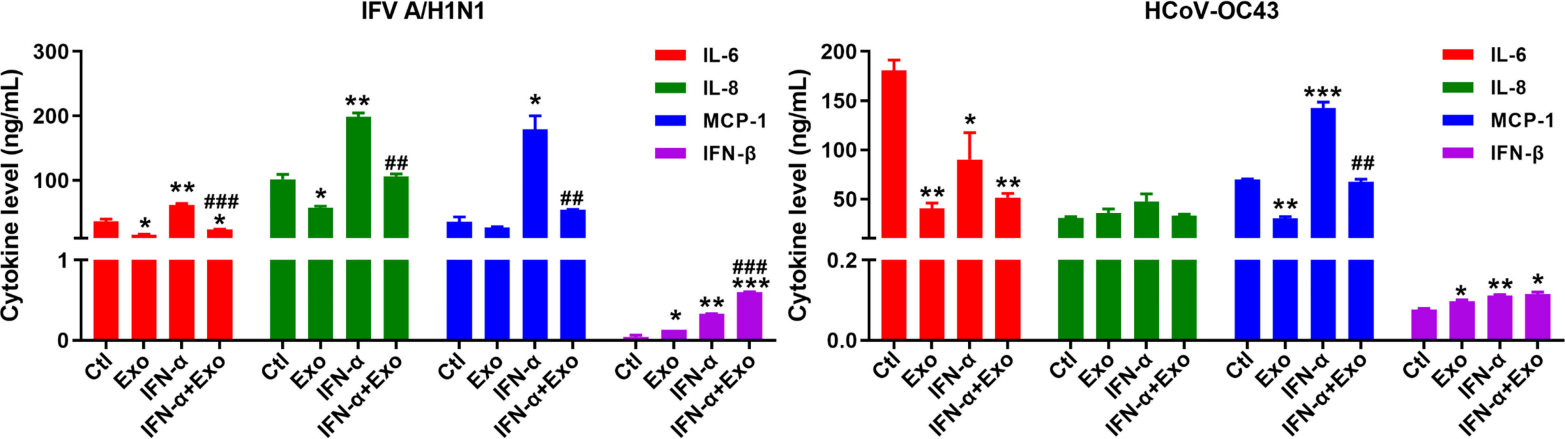
Anti-inflammatory effect of U-exo during respiratory virus infection of ALI culture. Apical side of fully differentiated primary ALI culture was inoculated with IFV A/H1N1, or HCoV-OC43 viruses along with either media control (Ctl) or U-exo (Exo), IFN-α (10 ng/mL), or IFN-α combined with U-exo (IFN-α+Exo) for 90 min. Media was removed from the apical side, and media containing U-exo and/or IFN-;α was added to the basolateral side. 6 days later, supernatants from the basolateral side were collected and ELISA assay was performed to measure the secretion levels of pro-inflammatory (IL-6, IL-8, and MCP-1) and antiviral (IFN-β) cytokines and chemokines. Data are shown as the mean ± standard deviation (SD) of the means from two independent experiments. Statistical analysis: *p < 0.05; **p < 0.01; ***p < 0.001, versus Ctl-treated cells. ^##^p < 0.01; ^###^p < 0.001, versus IFN-α-treated cells.

Consequently, we investigated the effect of U-exo on the level of cytokine secretion in the ALI culture model. The supernatants from basolateral side of IFV A/H1N1-, or HCoV-OC43-infected HNECs at 6 dpi were collected for the analysis of the major pro-inflammatory and antiviral cytokines and chemokines, IL-6, IL-8, MCP-1 and IFN-β (**Figure 7**). During IFV A/H1N1 infection, treatment with U-exo significantly reduced the secretion levels of all three cytokines, an observation in line with previous reports of the immunomodulatory effect of UCMSC (Chen et al., 2010). In contrast, IFN-α elevated IL-6, IL-8, and MCP-1 levels, and the pro-inflammatory effect of IFN-α was abrogated by the combined treatment of U-exo and IFN-α. Similarly, during HCoV-OC43 infection, U-exo treatment exhibited immunomodulatory function, showing a significantly reduced IL-6 and MCP-1 secretion levels compared with IFN-α-treated group. On the contrary, IFN-β secretion level was increased upon U-exo treatment, and a synergistic effect was observed upon the combined treatment with U-exo and IFN-α during IFV infection. These findings indicate the potential of U-exo to confer antiviral protection and to restrict the inflammatory response in human nasal epithelium.

## Discussion

While clinical studies involving the use of UCMSC- or MSC-derived exosomes are widely tested on various diseases, there are limited studies focused on the antiviral effects of U-exo. Our data demonstrated that U-exo carried out broadly effective antiviral activities against IFV and HCoV, as shown by the reduction in viral gene expression and viral titer in traditional submerged cell culture and ALI culture model. U-exo in combination with IFN-α exerted synergistically enhanced antiviral activity, whereas U-exo was able to modulate antiviral immune response *via* IFN activation. In addition, U-exo demonstrated an anti-inflammatory effect by attenuating pro-inflammatory cytokine release upon combined treatment with IFN-α as well as on the treatment of U-exo alone.

Exosomes serve as viral carriers, participate in the viral life cycle and contribute to control viral replication and pathogenesis (Shi et al., 2021). In this study, we introduce U-exo as a promising antiviral candidate that can confer therapeutic effects against respiratory viruses. Although the exact mechanisms of antiviral activity of U-exo are still unknown, our findings show that the antiviral effect of U-exo was abrogated when the exosomes were incubated with RNase prior to treatment. In conjunction with this result, we have previously identified small RNAs that inhibit replication and binding of Zika virus-infected UCMSCs (Seong et al., 2020). Furthermore, it is possible that U-exo may hinder viral replication process by degrading viral RNA. A study by Qian et al. revealed that U-exo inhibits the viral replication stage of HCV through functional miRNAs located within the exosomes (Qian et al., 2016). The main miRNAs responsible for the inhibitory action included let-7f, miR-145, miR-199a, and miR-221, all of which possessed binding sites in HCV RNA (Qian et al., 2016). In addition, U-exo exhibited a synergistic anti-HCV effect when combined with clinically approved HCV drugs, IFN-α and telaprevir (Qian et al., 2016). Therefore, it is likely that small RNAs inside U-exo have the potential to exert a broad antiviral effect on various types of viruses, including respiratory viruses. In correlation with Qian et al’s study, our small RNA-seq data of U-exo revealed that miR-125b-5p is one of the top differentially expressed miRNAs enclosed in sEVs of UCMSCs. Thus, we investigated whether miR-125b-5p is capable of modulating the expression of IFV genes. Interestingly, we found that miR-125b-5p was shown to exert potent antiviral activities against IFV possibly *via* the induction of ISGs. Thus, it will be interesting to further investigate the role of other miRNAs transferred *via* U-exo to target cells to reveal the detailed mechanisms for antiviral functions of exosome-derived small RNAs.

Considering the essential role of type I IFNs in limiting viral replication, we examined whether the combined treatment of U-exo and IFN-α enhanced IFN-mediated antiviral activities in the upper respiratory epithelium. We observed that U-exo restricted IFN-α induced pro-inflammatory cytokines while showing synergistic effects of attenuating viral replication. Also, U-exo may affect IFN-mediated signaling pathways and regulate the expression of ISGs, which are essential to restrict viral replication and prevent dissemination of the virus by inducing an antiviral state in nearby cells (Schneider et al., 2014). It is highly likely that U-exo upregulates Janus kinase/signal transduction and activator of transcription signaling pathways (JAK/STAT) that are required for optimal activation and function of ISGs. Thus, U-exo combined with IFN-α may stimulate antiviral effector mechanisms and thus contribute as a safe and effective therapeutic to treat respiratory infections.

UCMSCs have received recent attention due to their powerful regenerative and immunomodulatory potential, and their EVs provide an emerging role in modulating the symptoms of various diseases (Hollweck et al., 2012; Monguio-Tortajada et al., 2017; Lv et al., 2020; Liu et al., 2021; Palma et al., 2021). A recent study reported that co-culturing UCMSCs with IFV A/H5N1-infected alveolar epithelial cells significantly restored alveolar fluid clearance and protein permeability through the secretion of angiopoietin-1 and hepatocyte growth factor, factors which promote angiogenesis and cell growth and survival, respectively (Loy et al., 2019). In correlation with these studies, our study demonstrates that U-exo significantly suppressed secretion of pro-inflammatory cytokines in fully differentiated HNECs cultured at the ALI during respiratory viral infection. Thus, it will be interesting to further delineate U-exo-mediated activation of signaling pathways that induce the production of pro-inflammatory cytokines and chemokines.

A concerted effort has been made to develop physiologically relevant human airway cell culture models to study the pathogenesis of respiratory viruses and develop new therapeutics. The ALI culture system using primary HNECs has been recognized as an important research platform to study the pathogenesis of respiratory viruses, as nasal airway epithelial cells residing in the respiratory tract serve as the primary site of infection by respiratory viruses (Loo et al., 2020; Ahn et al., 2021). Loo et al. recently reported distinct replication kinetics of HCoVs using ALI culture model. According to the study, HCoV-OC43 replication was delayed peaking at 96 h after infection, which was similar to our data (Loo et al., 2020). Our findings show that HNECs cultured at the ALI were susceptible to infections with both IFV and HCoV, facilitating viral replication and recapitulating *in vivo* events like loss of tissue integrity and mucus-secreting goblet cells. As far as we know, our study is the first to utilize the ALI culture model to evaluate the antiviral activity of U-exo. Given that nasal epithelium is likely to be a key site of viral transmission and invasion, U-exo treatment intranasally could be highly effective in limiting respiratory viral spread.

In conclusion, our findings reveal a broad antiviral role of U-exo in the inhibition of viral replication and provide new insights into the development of broadly active antiviral agents to target respiratory viruses. In addition to studying the properties of natural exosomes, it will be important to engineer exosomes into highly optimized antiviral agents for therapeutic purposes. As an example, exosomes can be engineered to serve as a binding nano-decoy or drug delivery source (Popowski et al., 2021). A detailed understanding of the cellular response triggering the production of anti-viral exosomes may aid in the attempts to combat viral pathogens.

## Data Availability Statement

The raw data supporting the conclusions of this article will be made available by the authors, without undue reservation.

## Ethics Statement

The studies involving human participants were reviewed and approved by Korea University Medical Center Institutional Review Board. The patients/participants provided their written informed consent to participate in this study.

## Author Contributions

Conceptualization, S-JO, E-NL, OS; methodology, S-JO, E-NL, JL, J-HP; formal analysis, S-JO, E-NL, J-HP; investigation, S-JO, E-NL, JL, J-HP; resources, G-JC, I-HP; writing—original draft preparation, E-NL, OS; writing—review and editing, E-NL, S-JO, I-HP; visualization, I-HP, OS; supervision, I-HP, OS; project administration, I-HP, OS; funding acquisition, I-HP, OS. All authors have read and agreed to the published version of the manuscript.

## Funding

This research was funded by the Basic Science Research Program of the National Research Foundation of Korea (NRF) by the Ministry of Science, ICT & Future Planning (NRF-2019R1A2C1005961), Korea University Guro Hospital (KOREA RESEARCH-DRIVEN HOSPITAL) and grant funded by Korea University Medicine (K2117311).

## Conflict of Interest

The authors declare that the research was conducted in the absence of any commercial or financial relationships that could be construed as a potential conflict of interest.

## Publisher’s Note

All claims expressed in this article are solely those of the authors and do not necessarily represent those of their affiliated organizations, or those of the publisher, the editors and the reviewers. Any product that may be evaluated in this article, or claim that may be made by its manufacturer, is not guaranteed or endorsed by the publisher.

## Glossary

**Table:**

U-exo	small extracellular vesicles derived from human umbilical cord mesenchymal stem cells
IFV	human influenza A/B virus
HCoV	human seasonal coronavirus
IFN-α	interferon-alpha protein
ALI	air-liquid interface
SARS-CoV-2	severe acute respiratory syndrome coronavirus 2
MERS-CoV	middle east respiratory syndrome coronavirus
SARS-CoV-1	severe acute respiratory syndrome coronavirus 1
EVs	extracellular vesicles
miRNAs	microRNAs
MSCs	mesenchymal stem cells
HCV	Hepatitis C virus
UCMSCs	umbilical cord-derived mesenchymal stem cells
HNECs	primary human nasal epithelial cells
A549	human lung adenocarcinoma cells
HCT8	human colorectal adenocarcinoma cells
MRC5	human lung embryonic fibroblast cells
NS1	non-structural protein 1
IFV-PR8delNS1	Human influenza virus A/Puerto-Rico/8/34 PR8 lacking NS1
MOI	multiplicity of infection
TCID_50_	tissue culture infectious dose
hpi	hours post-infection
NTA	Nanoparticle Tracking Analysis
TEM	transmission electron microscopy
MTT	3-(4, 5-dimethylthiazolyl-2)-2,5-diphenyltetrazolium bromide
LDH	lactate dehydrogenase
GAPDH	Glyceraldehyde 3-phosphate dehydrogenase
DAPI	4,6-diamidino-2-phenylindole
TEER	trans-epithelial electrical resistance
H&E	hematoxylin and eosin
CM	Conditioned medium
HBV	Hepatitis B virus
sEVs	small EVs
RLR	retinoic acid-inducible gene I-like receptors
RIG-I	retinoic acid-inducible gene-I
MDA5	melanoma differentiation associated gene 5
MAVS	mitochondrial antiviral signaling protein
STING	stimulator of interferon response c-GAMP interactor 1
Mx1	MX dynamin like GTPase 1
ISG15	interferon-stimulated gene 15
OAS1	2’-5’-oligoadenylate synthetase 1
IFITM3	interferon induced transmembrane protein 3
IFN-β	interferon-beta
IFN-λ1	interferon-lambda 1
ISGs	interferon-stimulated genes
JAK/STAT	Janus kinase/signal transduction and activator of transcription signaling pathways
