# Supplementary material for: Anti-Viral Activities of Umbilical Cord Mesenchymal Stem Cell-Derived Small Extracellular Vesicles Against Human Respiratory Viruses
Source: Front Cell Infect Microbiol. 2022 Apr 21;12:850744. doi: 10.3389/fcimb.2022.850744 (PMC9085650; doi:10.3389/fcimb.2022.850744)
Supplement: Supplementary file 1 [file DataSheet_1.docx]

***Supplementary Material***

**
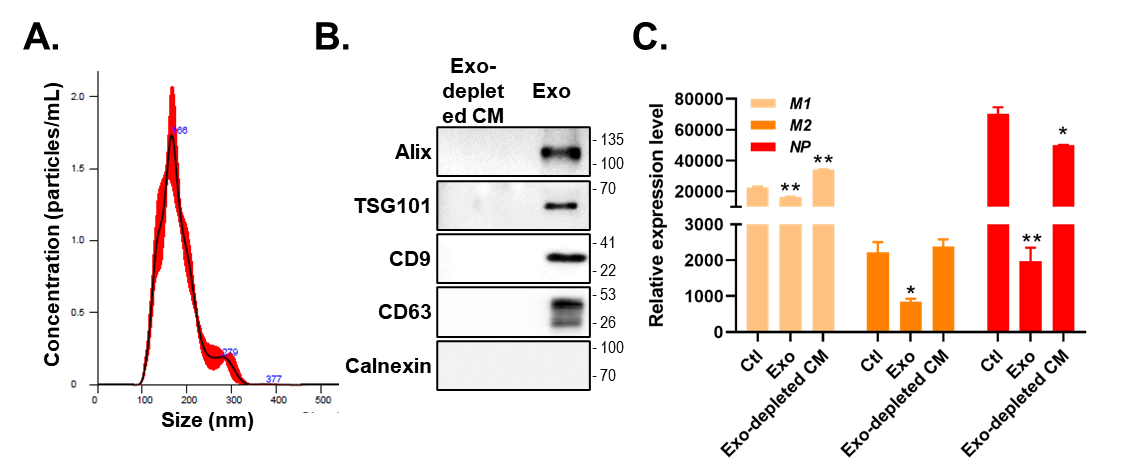
**

**Supplementary Figure 1. Isolation and characterization of umbilical cord mesenchymal stem cells (UCMSC)-derived exosomes (U-exo) using classical ultracentrifugation method.** UCMSC were cultured in medium containing exosome-depleted FBS for 48 h and culture supernatant was collected and centrifuged at 300 × g for 10 min to remove cellular debris and 0.22 μm filtration using syringe was used to discard small particles including apoptotic bodies. Subsequently, conditioned medium (CM) was collected for exosome isolation using the classical ultracentrifugation method. The supernatant was collected as exosome-depleted CM and pellet containing exosome was resuspended in PBS for subsequent nanoparticle tracking analysis (A). (B) Immunoblot analysis of U-exo markers (Alix, TSG101, CD9, and CD63) is shown. Calnexin (Endoplasmic reticulum marker) was used as a negative control. (C) A549 cells were infected with IFV A/H1N1 at an MOI of 1. After viral attachment, cells were treated with control (Ctl), U-exo (Exo) isolated by ultracentrifugation method for 48 h, after which viral gene expression levels were measured using RT-qPCR. Expression levels of the viral *matrix protein 1* (*M1*), *matrix protein 2* (*M2*) and *nucleoprotein* (*NP*) were quantified and normalized against *GAPDH*. (means ± SD; n = 3). Statistical analysis: *p < 0.05; **p < 0.01; versus Ctl-treated cells.


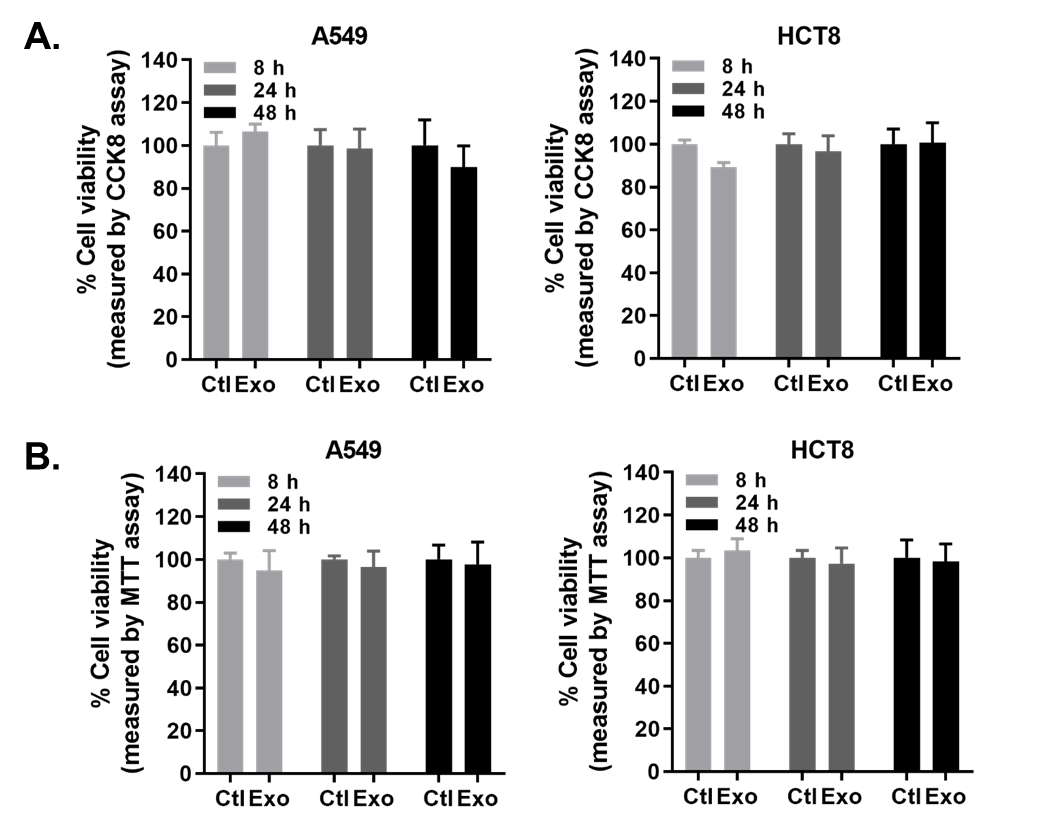


**Supplementary Figure 2. U-exo treatment in A549 and HCT8 cells does not cause cell cytotoxicity.** Cells were treated with medium (Ctl) or U-exo (Exo) for 8, 24, and 48 h. Cell lysates were used to measure cell viability via CCK8 and MTT assays. Values represent the mean and standard deviation of three independent experiments.

**
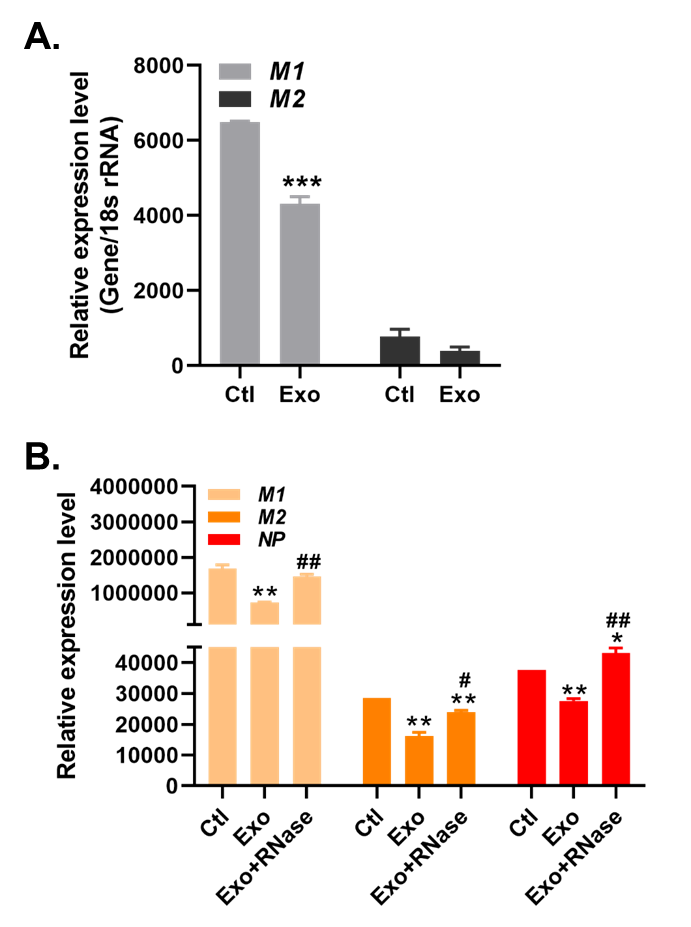
**

**Supplementary Figure 3. Antiviral role of U-exo treatment.** (A) A549 cells were infected with IFV A/H1N1 (MOI 1). After viral attachment, cells were treated with control (Ctl), U-exo (Exo) for 4 h, after which viral gene expression levels were measured using RT-qPCR. Expression levels of the *matrix protein 1* (*M1*) and *matrix protein 2* (*M2*) were determined and normalized against *18s rRNA*. (means ± SD; n = 3). Statistical analysis: *p < 0.05; ***p < 0.001, versus Ctl-treated cells. (B) A549 cells were infected with IFV A/H1N1 (MOI 1). U-exo were exposed to 1 μg/mL RNase for 1 h and added to IFV-infected cells. Viral gene expression levels were measured (means ± SD; n = 3). Statistical analysis: *p < 0.05; **p < 0.01; versus Ctl-treated cells. ^#^p < 0.05, ^##^p < 0.01, versus U-exo (Exo)-treated cells.


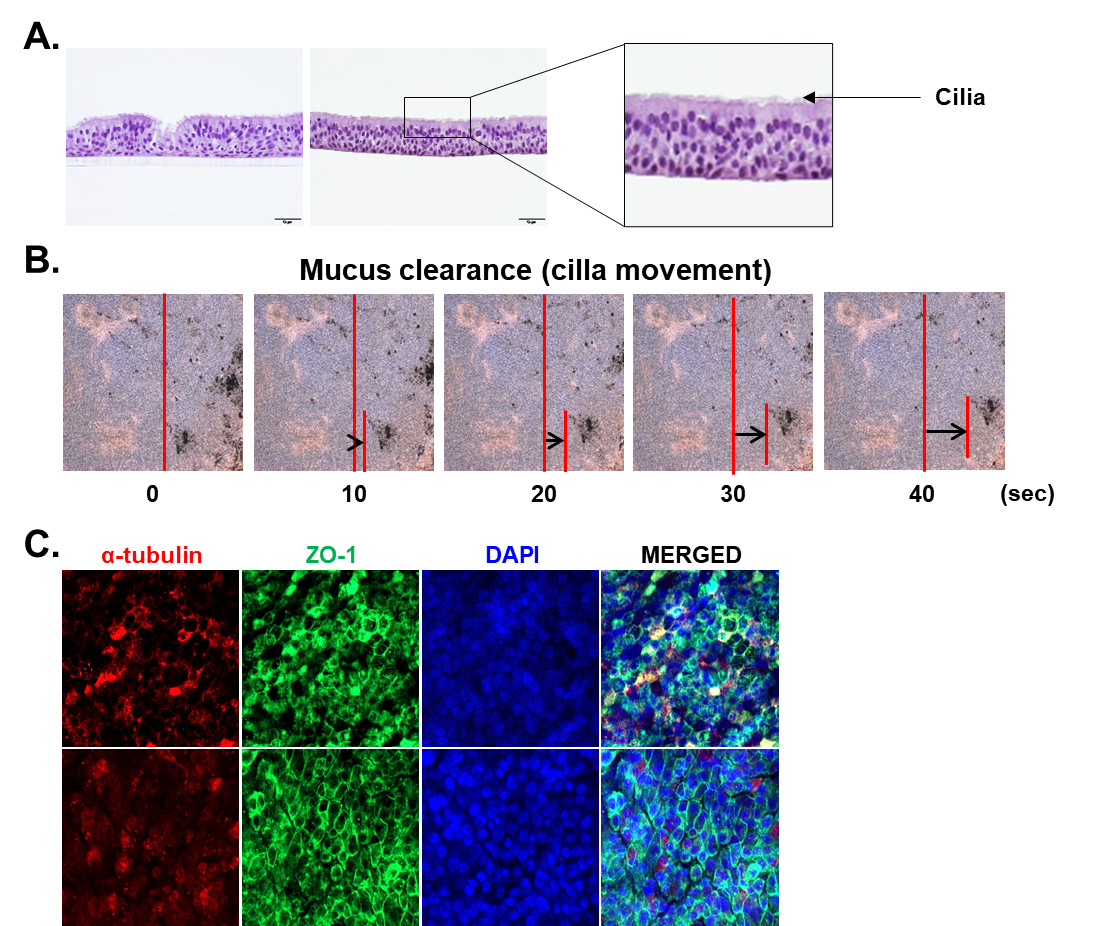


Supplementary Figure 4. Morphological evaluation and mucociliary function of human nasal epithelial cells (HNECs) cultured at the air-liquid interface (ALI). (A) The differentiation status of the HNECs cultured at the ALI were confirmed prior to their infection with IFV A/H1N1, or HCoV-OC43. After 3-weeks culturing, ALI culture inserts were fixed with 4% paraformaldehyde, and dehydrated in gradient alcohol series before they were embedded in paraffin. Sections of 4-μm thickness were subject for hematoxylin and eosin (H&E) staining. (B) To confirm mucociliary clearance, particulate matter (<10 μm, PM10) was added to surface of epithelial cells at upper chamber of ALI. Images of the cells were captured every 10 seconds. The ability of mucociliary clearance was confirmed by measuring the distance between the starting point of PM10 and the end point of PM10 after movement. (C) Structural integrity of the nasal epithelium at the ALI was evaluated by staining α-tubulin (red) and ZO-1 (green) and visualized by confocal microscopy. Scale bar = 20 μm.
